# Effect of human interference with maternal behaviour on development and blood biochemical parameters in the first 35 d of calves' life

**DOI:** 10.5194/aab-69-117-2026

**Published:** 2026-02-17

**Authors:** Marta Iwaszkiewicz, Aurelia Radzik-Rant, Katarzyna Czyż, Anna Wyrostek, Witold Rant

**Affiliations:** 1 Research and Education Station in Swojczyce, Wroclaw University of Environmental and Life Sciences, Wroclaw, 51-513, Poland; 2 Animal Breeding Department, Warsaw University of Life Sciences (SGGW), Warsaw, 02-786, Poland; 3 Institute of Animal Breeding, Wroclaw University of Environmental and Life Sciences, Wroclaw, 51-630, Poland

## Abstract

The purpose of this study was to determine the effect of interfering with the hair coat of newborn animals on their development and the efficiency of the immune system. The study involved 16 Holstein-Friesian calves divided into two groups (control and experimental). The animals from the control group were licked by the mother after birth, while these from the experimental group were handled after birth by a human. Weight and daily gains were determined, and blood was collected six times in 1 week intervals to analyse protein fractions as well as complete blood count. The concentrations of total protein, albumin, alpha and beta-globulins in the serum of calves from the experimental group did not differ from the control group. The level of gamma-globulins in the experimental group was lower at 14 and 21 d of age. The indices such as WBC, RBC, HGB, HTC, and PLT remained at similar levels in both groups and usually did not differ between the analysed periods in the studied groups. Only the content of platelets in both groups significantly increased from the seventh day of life compared to the baseline period. The levels of blood indices relating to the volume of the red blood cell and the mass and concentration of haemoglobin in it were not affected by interference with maternal behaviour, and changes in the values of these indices with age were noted. Based on the study, we concluded that the replacement of natural maternal care (licking) of a newborn calf by human action did not adversely affect the development and health of newborn calves, which confirms the validity of such a practice in dairy herds.

## 1 Introduction

Parturition, i.e. the transition from the intrauterine environment to the external environment, is the most difficult period in the life of a newborn, who has to face a change in environmental and nutritional conditions (Kirovski, 2015). The safe environment inside the mother changes to an environment where, in addition to lower temperatures, there are microorganisms attacking the body, and the energy intake changes from parenteral nutrition to the intake of food (including colostrum) by the newborn. Such great changes require adequate preparation and protection, for which the immune system is responsible (Stefaniak et al., 2012; Cortese, 2009; Gelsinger and Heinrichs, 2017). In addition to the immune system, protection from external environmental pathogens to the newborn can be provided by a mechanism that some researchers call the behavioural immune system (Gliński and Kostro, 2018). One mechanism of this system is licking of offspring, which is similar in all mammals (König von Borstel et al., 2010).

Immediately after birth, vigorously licking the newborn, the mother massages and activates all the nervous centres located in the medulla oblongata throughout the body. This activity is also intended to stimulate the newborn's activity, but most importantly, it leads to drying the newborn's body preventing heat loss. Moreover, it strengthens the bond between the mother and the newborn offspring (Kunowska-Slósarz and Różańska, 2009; Mandel and Nicol, 2017). This maternal behaviour increases in the newborn's ability to survive (Everett-Hincks and Dodds, 2008). The licking behaviour also allows the establishment of the bond between the mother and calf. Some evidences show that manual grooming of calves or cows with a brush can somehow mimic the stimulations observed when licking and thus cause a similar calming effect (Mandel and Nicol, 2017). This nursing behaviour is reflected in an increased chance of colostrum collection at the most optimal time (Kunowska-Slósarz and Różanska, 2009). By licking the offspring, mammals clean the neonate's body of residual water and foetal membranes, preventing the proliferation of bacteria on the body of the newborn. According to Gliński and Kostro (2018), a thin layer of saliva leaves a microfilm containing the bactericidal component of saliva: lysozyme. Lysozyme destroys bacteria by dissolving (lysing) the polysaccharide-peptide complex (peptidoglycan) composing bacterial cell wall. The bactericidal activity of lysozyme mainly targets gram-positive bacteria (Gajda and Bugla-Płoskońska, 2014). Lysozyme is also considered one of the main growth-limiting factors for intestinal bacteria (Bochnak et al., 2017).

In livestock herds, maternal ability resulting from behavioural traits is a very important feature (Yilmaz et al., 2011). Unfortunately, intensive herd improvement programmes often reduce the participation of mothers in caring for their offspring. Such examples can be seen in dairy cattle herds, where calves are often separated from their mothers immediately after birth and care is taken over by a human, whose role in this case is very important, as any activities performed must resemble the natural behaviour of the mother.

The purpose of the present study was to determine the effect of human interference with maternal behaviour (the licking stage) on the development and health of newborn calves by analysing the development of body weight, changes in protein fractions, and blood biochemical indices during the first 35 d of life. We hypothesized that artificial rearing of the newborn without mother contact, including a human intervention and increased hygienization, may have a negative effect on the newborn's development.

## 2 Materials and methods

### 2.1 Animals

The study was conducted in spring season on Holstein-Friesian calves kept at the Research and Education Station in Swojczyce, belonging to Wroclaw University of Environmental and Life Sciences, Poland. Sixteen heifer calves from cows maintained under standardized nutritional and environmental conditions and included in the same breeding programme were selected for the experiment. The sample size was determined on the basis of planned calving in the herd in a given period. The animals were selected for the study based on a normal course of delivery, without complications. The selected newborn calves with an average body weight of 34 kg showed normal vitality at birth. The calves were divided into two groups: control (C) and experimental (E), with eight animals in each group. All animals participating in the experiment stayed indoor, in the same room for the entire duration of the experiment.

After birth and vitality assessment, calves from the control group were put under the mother's muzzle and left to be licked for 30 min. After this time, they were weighed and placed in individual pens (dimensions 1.2×1.7 m, straw bedding). The calves in the experimental group, after the vitality assessment, were dried and massaged by the attendants, and the time taken to perform these activities was a total of 30 min. The calves were then weighed and, like the calves in the control group, were placed in separate pens. Newborn calves from both groups were given colostrum obtained from the mother in the amount of 2 L per head using a bucket with a nipple, and the time for its administration did not exceed 2 h from birth.

During the first 2 d, the calves were still given colostrum in the amount of 2 L three times a day. Over a period of the next 3 d, colostrum and further milk (2 and 3 L at day 35) were fed twice a day. Calves were given constant access to drinking water from day 2, and from day 5 solid feed was introduced in the form of a pre-starter mixture.

### 2.2 Research methods

The body weight of the tested calves in both groups was established by weighing the animals at 2, 7, 14, 21, 28, and 35 d of age, with an accuracy of 100 g. The stock scale for calves was used, and the calves were always weighed by the same two people. The growth rate of calves from both the control and experimental groups was also analysed by determining daily gains between the periods studied (g d^−1^).

Blood was collected from the jugular vein in the amount of 10 mL on the 2nd, 7th, 14th, 21st, 28th, and 35th days of life from calves of both groups. This period was chosen to demonstrate the trend in examined parameters levels. Blood was always collected in the morning (after the morning feeding) by the veterinarian, transported to the laboratory in refrigerated conditions, and then stored frozen until analysis. The calves were always restricted by the same person. Blood for morphological analysis was collected in tubes with EDTA (ethylenediaminetetraacetic acid), while to examine the proportion of protein fractions, it was collected in tubes with serum separation pellets.

To determine the biochemical parameters of blood, protein electrophoresis was performed at the “VetLab” laboratory (Wroclaw, Poland), allowing the determination of the content of protein fractions, i.e. albumin, globulins ( α , β , λ ), total protein content, and ratio of albumin to globulins.

Complete blood count was determined using a haematological analyser at the Veterinary Diagnostic Laboratory “Uni Lab” (Wroclaw, Poland). The following parameters were analysed: WBC (10^9^ L^−1^) white blood cells, RBC (10^12^ L^−1^) red blood cells, HGB (mmol L^−1^) haemoglobin content, HCT (L L^−1^) haematocrit, PLT (10^9^ L^−1^) platelet count, MCV (FL) mean corpuscular volume, MCH (F mol) mean corpuscular haemoglobin, and MCHC (mmol L^−1^) mean corpuscular haemoglobin concentration.

### 2.3 Statistical analyses

Statistical analyses were performed using Statistica 13.0 software (StatSoft Polska sp. z o.o., Poland). The results are presented as mean values and standard deviations. The Shapiro–Wilk test was used to analyse calf weight development to check normality of distribution. Differences in individual days between groups (C vs. E) were analysed by Student's t test. Tukey's test was used to compare differences within groups (C vs. E separately) between individual days, at a significance level of p<0.05 .

The following linear model, taking into account regression for body weight at birth, was used for body weight presentation:

1 $$Y_{ijk}=c_{i}+d_{j}+(cd{)}_{ij}+b(x-x_{\text{ś}r})+e_{ijk},$$

where $c_{i}$ is the group (control and experimental), $d_{j}$ is the age, $(cd{)}_{ij}$ is the interaction, b is the regression coefficient of analysed traits on weight at birth, $x-x_{\text{ś}r}$ is the deviation of the weight at birth of an individual from the average value, and $e_{ijk}$ is the residual error.

Normality of distribution in the analysis of proteins and blood indices was checked using the Shapiro–Wilk test. In the absence of a normal distribution, differences in individual days between groups (C vs E) were analysed using the Mann–Whitney U test for independent samples; in the case of a normal distribution, the Student's t test was used. The assumed significance level was p<0.05 .

## 3 Results

### 3.1 Effect of maternal behavioural interference on body weight development and daily gains of calves 

Analysing the body weights of calves, it can be concluded that heifers from the experimental group reached a numerically higher body weight than heifers from the control group. Significant differences in the level of this parameter between the studied groups were only noted on 21st and 28th days of life ( P<0.05 ; Table 1).

**Table 1:** Body weights of calves from the experimental and control groups (kg). Bold values indicates significant differences.

Day	Body weight (mean value)		$S_{E}$	p value
	Control group	Experimental group		
2	35.88	36.20	0.22	0.32
7	39.05	39.07	0.09	0.91
14	42.14	42.05	0.08	0.48
**21**	**44.81**	**45.15**	**0.07**	**0.01**
**28**	**48.31**	**48.70**	**0.10**	**0.02**
35	51.43	51.93	0.17	0.06

In contrast, slightly higher, but not statistically significant, daily gains were found in calves in the control group during the second week of life. Lower daily gains ( P<0.05 ) were recorded in the control group between the 14th and 21st days of life, after which the growth rate levelled off by the end of the experiment in both study groups (Table 2).

**Table 2:** Average weight gains of calves from the experimental and control groups (g d^−1^). Bold values indicates significant differences.

Period (d)	Body weight gain (mean value)		$S_{E}$	p value
	Control group	Experimental group		
2–7	603.36	606.64	18.74	0.95
8–14	440.12	425.95	6.61	0.16
**15–21**	**382.17**	**442.83**	**6.08**	**0.01**
22–28	500.25	506.89	9.74	0.64
29–35	445.77	461.37	15.64	0.50
2–35	466.52	481.59	5.13	0.06

### 3.2 Effect of maternal behavioural interference on plasma protein levels 

**Figure 1:**
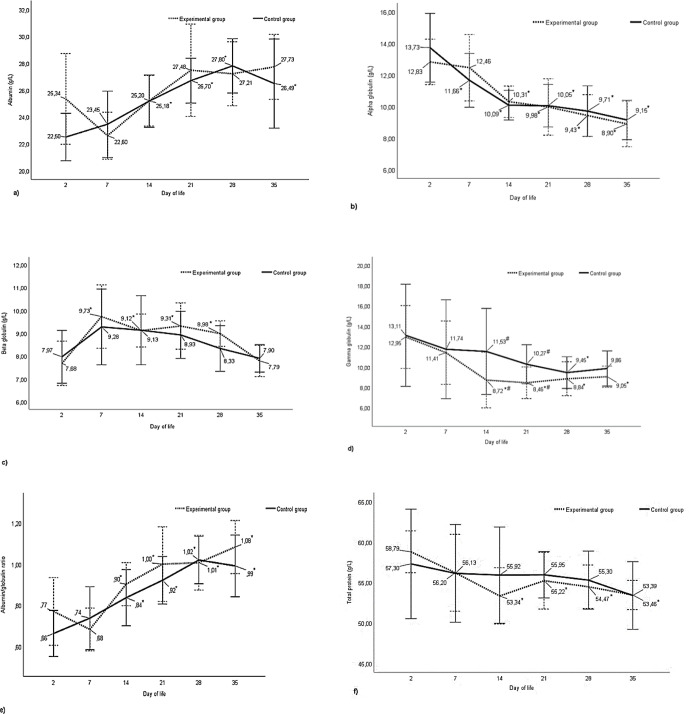
Changes in plasma protein content in the studied groups of calves from 2 to 35 d of age **(a)** albumins, **(b)** alpha-globulins, **(c)** beta-globulins, **(d)** gamma-globulins, **(e)** albumin/globulin, and **(f)** total protein. ^*^ – differences statistically significant in relation to the first sampling, ^*#*^ – differences statistically significant between the control and experimental group of calves.

No statistically significant differences were observed in albumin content between the experimental and control groups during the analysed periods from day 2 after birth to day 35 of calves' life. In the experimental group, in contrast to the control group, a decrease in albumin content was observed on day 7 of life compared to the initial study period (day 2) and a subsequent increase, although not statistically confirmed, in their level between day 15 and 21. In the subsequent study periods, the level of this fraction stabilized. In the control group, the calves' blood albumin content increased linearly throughout the study period and was higher ( P<0.05 ) at 21, 28, and 35 d of age compared to the baseline period (Fig. 1a).

There were no statistically significant differences between the control and experimental groups on all days of life studied in the level of alpha-globulins in the blood of calves (Fig. 1b). The content of this protein fraction reached its highest value on day 2 after birth, after which it decreased with the age of the animals in both groups. A decrease in the content of this fraction ( P<0.05 ) compared to baseline was registered as early as day 7 in the control group and on day 14 in the experimental group, and it persisted until the end of the study. The difference between the lowest level of alpha-globulin fraction in the control and experimental groups on day 35 and the highest on day 2 after birth was about 34 % and about 30 %, respectively (Fig. 1b).

As with alpha-globulins, there were no statistically significant differences between the control and experimental groups in the content of beta-globulins at any of the analysed periods (Fig. 1c). The level of the content of this protein fraction was similar in both groups during the initial study period, with an increase recorded at day 7 of life. In subsequent periods, the content of beta-globulins decreased. In the control group, the decrease was linear with a mild course, but the differences in the content of beta-globulins between the days of life studied were not statistically confirmed. In the experimental group, significant differences in the content of these proteins were recorded at 7, 14, and 21 d of age in relation to the initial period. For both groups, the lowest values in the level of beta-globulins were observed at 2 and 35 d of life and were approximately 21 % and 14 % lower in the experimental and control groups, respectively, compared to the maximum values obtained at 7 d of life (Fig. 1c).

Analysis of gamma-globulin content showed statistically significant differences between the control and experimental groups. The content of this fraction of proteins in the blood of calves from the experimental group at 14 and 21 d of their life was lower ( P<0.05 ) compared to calves in the control group (Fig. 1d). Considering the course of changes in the level of this fraction of proteins at different periods, it can be observed that the content of gamma-globulins in both groups shows a decreasing trend and again increasing from day 21 in the experimental group and from day 28 in the control group. In the experimental group, the lowest content of this fraction was registered on day 21, which was about 35 % lower ( P<0.05 ) than the content registered on day 2 after birth. Smaller ( P<0.05 ) values of this protein in this group were also recorded at 14, 28, and 35 d compared to the initial period. In contrast, in the control group, the reduction in gamma-globulin content was milder, and the difference ( P<0.05 ) between the level on day 28 (the lowest value) and day 2 after birth was about 27 % (Fig. 1d).

The above results are reflected in the ratio of albumin to globulin fractions in calf blood, as shown in Fig. 1e. No statistically significant differences were observed between the control and experimental groups at any period studied. In both groups, the albumin/globulin ratio showed an increasing trend with age. Differences ( P<0.05 ) between the initial period and the other study periods were registered from the 14th day of calves' life in the control and experimental groups.

Considering the content of total protein in the blood of the tested animals, there were no statistically confirmed differences between calves licked by the mother ( K ) and calves subjected to human intervention ( D ). The level of this parameter decreased with the duration of the experiment only in the experimental group. In this group, the total protein content of calves' blood on day 2 after birth was higher ( P<0.05 ) than on days 14, 21, 28, and 35 (Fig. 1f).

### 3.3 Effect of interference with maternal behavioural on the level of blood biochemical parameters

Analysing the biochemical parameters of calves' blood (white blood cells – WBC, red blood cells – RBC, haemoglobin – HGB, haematocrit – HCT, platelet – PLT, mean corpuscular volume – MCV, mean corpuscular haemoglobin – MCH, mean corpuscular haemoglobin concentration – MCHC), there was no significant effect of human interference with maternal behaviour on their levels in all periods studied (Table 3). For most of the blood indices studied, no differences within groups were also registered between days of observation. WBC levels were higher in the middle observation period, i.e. days 14 and 21, compared to the initial period in both groups, but the differences were not statistically confirmed. As in the case of leukocytes, statistically significant differences were not observed in the level of red blood cells (RBC) between the groups of calves studied as well as the periods, the values of this parameter being equalized throughout the experiment. The content of haemoglobin (HGB) and haematocrit (HCT) in the blood of calves from both the control and experimental groups was also at similar levels regardless of interference with maternal behaviour and the day of observation within the groups.

Analysing the platelet level (PLT), a lower ( p<0.05 ) value of this indicator was observed in both study groups at day 2 after birth compared to 14, 21, 28, and 35 d of observation in the control group and compared to 7, 14, and 21 d of life in the experimental group (Table 3). Another parameter analysed was mean red blood cell volume (MCV). In the case of this parameter, interference with the maternal behaviour of the tested calves also had no effect ( p>0.05 ) on its level on any day of observation. In both groups, a decrease in the value ( p<0.05 ) of MCV was observed from day 14 to the end of the experiment compared to baseline (Table 3).

Mean red blood cell haemoglobin mass (MCH) was similar in both study groups and all periods from 2 to 35 d of age. In contrast, the value of mean haemoglobin per unit red blood cell volume (MCHC) increased with age in both groups. In the control group, the value of MCHC was higher ( p<0.05 ) at 21, 28, and 35 d of observation compared to 2 d of life, and in the experimental group ( p<0.05 ) at 28 and 35 d with respect to baseline (Table 3). In this case, no statistically significant differences were observed between the study groups at different periods.

**Table 3:** Changes in the level of blood biochemical parameters in the studied groups of calves from 2 to 35 d of age (mean values ± standard deviation).

Parameter	Group	Day of life					
		2	7	14	21	28	35
WBC (10^9^ L^−1^)	C	8.69 ± 2.13	9.46 ± 4.67	11.08 ± 3.57	9.59 ± 2.06	9.89 ± 2.09	9.15 ± 3.56
	E	7.98 ± 2.24	7.94 ± 2.13	10.67 ± 3.10	8.71 ± 1.67	8.66 ± 2.03	7.36 ± 1.51
RBC (10^12^ L^−1^)	C	7.24 ± 0.96	6.99 ± 0.87	6.99 ± 0.83	6.74 ± 1.38	7.73 ± 1.52	7.42 ± 1.21
	E	7.39 ± 1.03	7.32 ± 1.11	7.14 ± 0.95	7.29 ± 1.25	7.10 ± 1.97	7.45 ± 0.71
HGB (mmol L^−1^)	C	5.58 ± 0.70	5.29 ± 0.70	5.21 ± 0.64	5.09 ± 0.89	5.39 ± 0.73	5.49 ± 0.84
	E	5.60 ± 0.67	5.58 ± 0.83	5.34 ± 0.66	5.30 ± 0.75	5.41 ± 0.79	5.43 ± 0.53
HCT (L L^−1^)	C	0.30 ± 0.04	0.27 ± 0.04	0.26 ± 0.03	0.25 ± 0.06	0.24 ± 0.07	0.26 ± 0.05
	E	0.31 ± 0.05	0.29 ± 0.05	0.27 ± 0.04	0.27 ± 0.05	0.27 ± 0.04	0.26 ± 0.03
PLT (10^9^ L^−1^)	C	447.13^a^ ± 137.30	796.62 ± 115.45	1019.88^b^ ± 214.33	922.75^b^ ± 173.74	948.25^b^ ± 365.68	869.13^b^ ± 278.76
	E	447.88^a^ ± 136.36	834.13^b^ ± 174.64	957.00^b^ ± 190.57	823.25^b^ ± 193.53	813.75 ± 337.62	764.25 ± 227.10
MCV (FL)	C	41.00^a^ ± 2.39	38.83 ± 2.48	37.50^b^ ± 2.20	35.63^b^ ± 2.33	34.75^b^ ± 2.19	34.50^b^ ± 1.51
	E	41.38^a^ ± 2.00	40.88 ± 2.70	37.63^b^ ± 0.92	36.50^b^ ± 1.20	34.88^b^ ± 1.89	34.75^b^ ± 0.89
MCH (f mol)	C	0.77 ± 0.06	0.76 ± 0.04	0.75 ± 0.03	0.76 ± 0.03	0.75 ± 0.02	0.74 ± 0.03
	E	0.76 ± 0.03	0.76 ± 0.03	0.75 ± 0.02	0.73 ± 0.03	0.72 ± 0.01	0.72 ± 0.02
MCHC (mmol L^−1^)	C	18.85^a^ ± 0.89	19.40 ± 0.34	19.84 ± 0.46	20.92^b^ ± 1.17	21.46^b^ ± 1.48	21.56^b^ ± 1.29
	E	18.59^a^ ± 0.76	19.19 ± 0.48	19.88 ± 0.46	19.98 ± 0.97	20.61^b^ ± 0.99	20.71^b^ ± 1.00

^a, b^ Differences statistically significant in relation to the first sampling; WBC – white blood cells, RBC – red blood cells, HGB – haemoglobin, HCT – haematocrit, PLT – platelet count, MCV – mean corpuscular volume, MCH – mean corpuscular haemoglobin, MCHC – mean corpuscular haemoglobin concentration.

## 4 Discussion

### 4.1 Body weight development and daily gains in calves 

The birth weight of calves is one of the main determinants of neonatal survival, their subsequent health, and, consequently, profitability in breeding (Kupczyński and Adamski, 2004). In the present study, the average weight of heifer calves at birth (Table 1) was lower than that expected for heifers of this breed, which should oscillate around 38 kg (Król and Jawor, 2019). Numerically higher birth weights for calves of this breed compared to the present study were also obtained by Samolovac et al. (2020). Numerically lower body weights of calves from the group licked by the mother on all test days, except day 35, should be taken with caution and may have been due to the lower birth weights of heifers in this group, rather than the different treatment of the animals immediately after birth.

Average daily calf gains in both study groups were at similar levels, except days 15–21, when the gain was significantly higher in the experimental group (Table 2). The decrease in gains (up to 390 g d^−1^) noted in this study only in the control group between 15 and 21 d of age was similar to the study by Kisac et al. (2011), who analysed calf development in relation to maternal age.

### 4.2 Plasma protein levels in the calves 

Analysed changes in the serum protein content of calves from 2 d after birth to 35 d of age did not indicate significant differences between the experimental and control groups. More differences were found in the change in the content of individual protein fractions (albumin, alpha, beta- and gamma-globulins, and total protein) depending on the study period.

Human interference with maternal behaviour had no effect on the level of albumin in the blood of calves during all study periods (Fig. 1a). The content of this fraction, the main protein required to regulate and maintain osmotic pressure, necessary for the proper distribution of body fluids in the vascular system and tissues, was at or below the limit of reference values and did not exceed 30 g L^−1^, both in the control and experimental groups but showed an increasing trend in both groups, especially from day 21 to the end of the observation period (Fig. 1a; Marc et al., 2018; Winnicka, 2021). An increase in albumin content in calves during the first month of life was also recorded by Knowles et al. (2000), Ježek et al. (2006), Piccione et al. (2009), and Tóthová et al. (2016). The lower content of albumin immediately after birth is associated with a higher content of globulins, whose higher blood concentration is associated with colostrum consumption (Thomas, 2000). Changes in albumin concentration during the first period of life reflect the average half-life of albumin, which in ruminants is 14 to 16 d, after which the liver is responsible for albumin synthesis (Lassen, 2004; Thrall et al., 2004). At a later stage, the concentration of albumin in the body of growing animals is influenced not only by age but also by nutrition (Kaneko, 1997).

The dynamics of changes in globulin fractions, especially alpha- and beta-globulins in the calves studied, were similar. The content of these globulin fractions, as in the case of albumin, was not affected by human interference with maternal behaviour (Fig. 1b, c). The highest concentration of alpha-globulins was registered on day 2 after birth in calves in both study groups, after which the concentration decreased until the end of the observation period. On the other hand, the content of beta-globulins in the blood serum of the studied calves on day 2 after birth was the lowest and then increased on day 7. A decrease in the content of alpha-globulins, an increase in the concentration of beta-globulins on day 7, and a subsequent decrease in the level of this fraction were also recorded by Tóthová et al. (2016) and Piccione et al. (2009) in calves in the first month of life. Similarly, as in the present study, Szewczuk et al. (2011) found higher concentrations of alpha-globulins in calves at day 5 compared to values measured at 30 d of age.

According to Kaneko (1997), the high amount of alpha-globulins in the blood serum of neonatal and young animals is due to the presence of higher concentrations of certain proteins from this fraction, which must protect young animals from pathogen attack during the first period of life. Many acute-phase proteins migrate into the regions of alpha-globulin (haptoglobin, ceruloplasmin, α 1-acid glycoprotein, α 1-antitrypsin), making the increase in this fraction possibly related to inflammation and stimulation of various stress factors (Kaneko, 1997). However, higher concentrations of alpha-globulins in calves after birth are not necessarily a sign of activation of inflammatory processes or a sign of disease. Lomborg et al. (2008) indicate that not only inflammatory stimuli but also environmental conditions can trigger changes in the content of acute-phase proteins and that increases in alpha-globulin concentrations can occur in conditions unrelated to inflammatory diseases. On the other hand, the recorded increase in the content of beta-globulins in the postnatal period from day 7 onward with respect to the initial period may have been related to an increase in some proteins, especially complement, from this fraction, which, like acute-phase proteins, are involved in the response to environmental stress (Bernabucci et al., 2009). Changes in the content of the above-described protein fractions were not related to the licking process or the replacement of this process by a human.

In contrast to the protein fractions discussed above, the gamma-globulin content in the blood of calves was affected ( P<0.05 ) by human interference in maternal behaviour. While on the second and seventh day after birth, the level of gamma-globulin content in both groups of calves studied was similar, already on days 14 and 21 the concentration of this protein was lower ( P<0.05 ) in the group of calves subjected to human postnatal activities (Fig. 1d). In this group of calves, gamma-globulin concentrations began to increase again from day 28, which may indicate an increase in the activity of the body's own immune system and a transition from passive to active immunity (Meyer and Harlay, 2004). In the control group, the lowest levels of this protein were recorded at day 28 and 35 compared to earlier study periods, which may indicate that the newborn calf licked by its mother has been benefiting from colostrum immunity for a longer period of time (Fig. 1d). A slow decrease in this fraction in the first month of life in the blood of calves was also noted in the study by Tóthová et al. (2016). The changes in gamma-globulin content can be attributed to the degradation of colostrum-derived immunoglobulins and the slow, gradual initiation of production of the calves' own immunoglobulins by the maturing immune system.

Changes in the concentration of the analysed albumin and globulin fractions in newborn calves during the first month of life are also reflected in the ratios of albumin to globulin. The value of this ratio increased with age (Fig. 1e). The lower value of this parameter in the first 2 weeks of calves' life indicates a higher proportion of globulins in the serum of these animals, which give way to an increasing proportion of albumin. According to Alberghina et al. (2010), the albumin/globulin ratio should be interpreted with caution, noting which component of this ratio has changed.

The concentration of individual protein fractions shapes the picture of total protein in the blood. As in the case of the albumin and globulin fractions analysed, except for gamma-globulins in the calves studied, human interference with maternal behaviour also did not affect the concentration of total protein in serum. No statistically significant changes in protein content were also recorded for the experimental periods studied in licked calves. In calves subjected to human postpartum activities, the total protein content decreased compared to the baseline period starting at 14 days of age (Fig. 1f). No changes in total protein concentration during the neonatal period in calves were recorded in their studies by Piccione et al. (2009) and Tóthová et al. (2016). According to Thrall et al. (2004), modifications in total protein concentrations can result from changes in the concentrations of albumin, globulins, or both fractions, but increased concentrations of these do not always result in a detectable increase in total protein concentration. In the present study, despite the lack of statistical differences in the control group, a trend towards a decrease with age can be noted (Fig. 1f). Hammon et al. (2002) report that immediately after birth, serum protein concentrations in most animals are quite low due to minimal amounts of immunoglobulins, with an increase occurring during the first 24 h of life, reflecting intestinal absorption of proteins (especially immunoglobulins) from colostrum due to an increased intestinal permeability. In the present study, also the highest concentration of total protein in the blood of calves was registered on day 2, that is, already after colostrum administration and globulin intake by the suckling calves. The content of total protein in the blood serum of the studied calves, regardless of interference with maternal behaviour and the study period, was in the range of 53.39–58.79 g L^−1^ and was within the reference limits (51–71 g L^−1^) for this parameter (Winnicka, 2021).

Changes in the electrophoretic profile of serum proteins may indicate diseases or inflammatory processes in the body, but these changes during the first 35 d of life of newborn animals may also be related to physiological changes and adaptive processes.

### 4.3 Levels of blood biochemical indices in the calves 

Haematological and biochemical indicators in the blood of production animals allow not only monitoring of their health and early diagnosis of disease states but also evaluation of physiological changes occurring from birth to the achievement of body stability (Mohri et al., 2007; Dillane et al., 2018; Golbeck et al., 2019).

Analysis of biochemical parameters of the blood of calves from birth to 35 d of age showed that interference with maternal behaviour did not affect the level of haematological indices. Larger changes were noticed within the groups depending on the study period.

The obtained values of the index of white blood cells in the studied calves were within the reference norms provided for adult animals (4.0–1 $2.0\times 10^{9}$ L^−1^; Winnicka, 2021). Song et al. (2020) indicate a high correlation of WBC with the increase of neutrophils, which is an extremely valuable tool in the assessment of immunity and the diagnosis of diseases in young animals, but the level of these blood components was not analysed in the present study. Variability in the number of WBCs in the blood of young organisms is indicated by Ježek et al. (2011), attributing it to the different lifespan of different types of leukocytes. Variability in the value of this indicator in the first 35 d of life, although not statistically confirmed, was also observed in the present study (Table 3).

The level of red blood cells (RBC) in both the experimental and control groups of the studied calves slightly exceeded the reference values (5.0– $7.0\times 10^{12}$ L^−1^; Winnicka, 2021; Table 3). In general, this parameter remained stable throughout the study period, which was also noted by Knowles et al. (2000) when analysing changes in biochemical parameters in the blood of calves during the first month of life. Slightly higher values compared to those obtained in the present experiment and high dynamics of RBC changes were obtained by Golbeck et al. (2019) in newborn Holstein-Friesian calves. Changes in RBC content, according to many authors, may be due to the amount of erythrocyte production at birth and the shorter life span of intrauterine-produced erythrocytes (Brun-Hansen et al., 2006; Ježek et al., 2011).

There was no effect of either interference with maternal behaviour or the study period within the groups on the level of haemoglobin (HGB) in the calves' blood. The value of this indicator was stable, within the reference values for the species, throughout the observation period (Table 3). Haemoglobin values in the blood of calves oscillated between 5.09 and 5.60 mmol L^−1^ in both groups, and were closer to the lower limit of reference norms for cattle (4.96–8.69 mmol L^−1^) (Winnicka, 2021). The reason for the decrease in haemoglobin in the blood may be a deficiency of certain amino acids, which cannot be linked to postpartum activities performed by humans in the experimental group, especially since this indicator took on similar values in the blood of calves licked by their mothers. Significantly higher values of haemoglobin in the blood of calves reaching from 9 to over 12 g dl^−1^ were indicated by Song et al. (2020).

The haematocrit values obtained in both groups of calves are within the reference limits (0.24–0.46 L L^−1^; Winnicka, 2021). Despite the lack of significant differences in the magnitude of this indicator between the studied groups of these animals, it is noticeable that its level is lower – also not statistically confirmed – in the final period of the experiment in relation to day 2 after birth (Table 3). A similar decrease in the level of haematocrit in the first weeks of life of calves was recorded in the study by Knowles et al. (2000).

Platelets are the only parameter among the analysed blood biochemical indicators that significantly exceeded the level of reference values. The value of this parameter in the blood of calves in the initial research period was within the reference values (150– $650\times 10^{9}$ L^−1^); however, already in the first week of life of these animals it increased ( p<0.05 ) almost twice, and the peak level of PLT was reached in the second week of life of calves. In their studies, many researchers have indicated that a significant increase in PLT levels in the first weeks of a calf's life is a fairly commonly observed phenomenon (Knowles et al., 2000; Brun-Hansen et al., 2006). The increase in platelet counts in newborn calves may be a result of their accumulation around the umbilicus, as these blood elements are essential for the healing of the ruptured umbilical cord (Strous et al., 2021).

The analysed parameters included in the haematological profile of blood also include those that indicate the volume of the red blood cell (MCV) and the mass and concentration of haemoglobin in the red blood cell in the animals (MCH and MCHC). In our study on calves' blood, no effect of interference with maternal behaviour on the value of the above-mentioned indicators was registered. MCV values in the blood of calves were within the reference norms (40–60 fl) only on the second day after birth. The values obtained during the other study periods were lower ( p<0.05 ) and remained below the reference norms in both groups (Table 3). According to Constable et al. (2017), the reduction in red blood cell volume during the first period of life is due to the replacement of red blood cells containing foetal haemoglobin with smaller erythrocytes containing haemoglobin A. This suggests that a decrease in MCV in newborn calves is a physiological process and should not be taken as a sign of pathological changes, such as anaemia (Golbeck et al., 2019). The values of these indices in young animals may be different from those in adult animals, i.e. they may not always conform to the reference values set for the latter.

The values of biochemical parameters indicating the saturation of blood cells with haemoglobin demonstrated a stabilization between the studied periods in the blood of calves with regard to MCH and an increase in values ( p<0.05 ) with age in the case of MCHC. This result may have been influenced by the high individual variability of newborn calves (Golbeck et al., 2019). These dynamics of change registered in the indicators discussed above may have been dictated by the process of foetal blood degradation and replacement (Burn-Hansen et al., 2006).

## 5 Conclusions

Human interference with maternal behaviour did not affect the values of parameters examined of newborn calves analysed up to 35 d of age. Growth rates between calves licked by their mothers and calves subjected to human care mostly remained at similar levels and did not differ from those obtained on production farms.

The levels of total protein, albumin, and alpha- and beta-globulins in the serum of calves subjected to human care after birth did not differ from those of licked calves. Only the level of gamma-globulins in the group of human-treated calves was lower at 14 and 21 d of age compared to maternally licked calves, which may indicate a faster activation of the animals' own immune system in these animals.

In the plasma of calves, the content of the protein fractions studied changed depending on the study period. The content of albumin increased with the age of newborn calves, while globulin fractions decreased or slightly increased again in accordance with physiological and adaptive changes. Following the changes in albumin and globulin levels, the value of the albumin/globulin ratio increased. Plasma total protein levels did not differ between 2, 7, 14, 21, 28, and 35 d of age.

In the blood of calves, biochemical indices such as WBC, RBC, HGB, HTC, and PLT remained at similar levels in individuals both subjected to human care at birth and licking by the mother, and mostly did not differ in content between the analysed periods within the studied groups. Only the content of platelets in both groups of calves significantly increased from the seventh day of life compared to the baseline period. The levels of blood indices relating to the volume of the red blood cell and the mass and concentration of haemoglobin in it, i.e. MCV, MCH, MCHC, in the calves studied were not affected by interference with maternal behaviour, and changes in the values of these indices with age were due to the degradation and replacement of foetal blood.

In summary, the replacement of natural maternal care (licking) of a newborn calf by human action did not adversely affect the development and health of newborn calves, which confirms the validity of such a practice in dairy herds. The limitation of our study is the relatively small sample size, as well as a lack of behavioural observations and other welfare measures analyses that would strengthen the conclusions.

## Data Availability

All the data generated or analysed during this study are included in this published article.
